# In Case of an Emergency: The Development and Effects of a Digital Intervention for Coping With Distress in Norway During the COVID-19 Pandemic

**DOI:** 10.3389/fpsyg.2021.705383

**Published:** 2021-11-22

**Authors:** Tine Nordgreen, Emilie Sektnan Nordby, Sunniva Burok Myklebost, Eivind Flobak, Smiti Kahlon

**Affiliations:** ^1^Division of Psychiatry, Haukeland University Hospital, Bergen, Norway; ^2^Department of Global Public Health and Primary Care, Faculty of Medicine, University of Bergen, Bergen, Norway; ^3^Department of Biological and Medical Psychology, Faculty of Psychology, University of Bergen, Bergen, Norway; ^4^Department of Information Science and Media Studies, Faculty of Social Sciences, University of Bergen, Bergen, Norway

**Keywords:** self-guided Internet-delivered intervention, COVID-19, positive psychology, cognitive behavioral therapy, person-based approach

## Abstract

**Background:** The COVID-19 pandemic and its consequences has been found to negatively affect the general population’s psychological well-being.

**Objective:** The objectives of this paper are to report on the development and clinical effects of a self-guided Internet-delivered intervention for adults in Norway who suffer from mild to moderate psychological distress during the COVID-19 pandemic.

**Methods:** The participants, recruited between April and December 2020, were randomized to receive a new treatment module either every third or every fifth day. The clinical outcomes were self-reported depressive and anxiety symptoms and change in positive and negative emotions.

**Results:** A total of 1256 individuals accessed the pre-screening survey, 407 were eligible and 92 provided contact information, where 82 were included in the study, *n* = 44 in the 3-day group and *n* = 38 in the 5-day group. Overall, the statistical analyses showed a significant decrease in depressive and anxiety symptoms and an increase in positive emotions, with small and moderate within group effect sizes. No significant differences between the groups were identified in clinical outcomes or adherence.

**Conclusion:** These findings indicate that psychological distress in the general population during the COVID-19 pandemic may be reduced through the use of a scalable self-guided Internet-delivered intervention. Furthermore, the lack of significant differences between the 5-day and 3-day group may indicate that the intervention can be delivered at a more intensive pace without negatively affecting treatment outcomes. The results need to be interpreted with caution as the sample was self-selected, as well as the lack of passive control group. Hence the results may be attributed to external factors.

## Introduction

The COVID-19 pandemic and its consequences has been found to negatively impact mental well-being (Wang et al., 2020). Since the start of the pandemic, there has been an increase in symptoms of depression, anxiety, and stress in the general population (Wang et al., 2020). As such, there is a need for effective and scalable psychological interventions for the general population with the aim to increase coping and reduce the risk for anxiety and depression (Holmes et al., 2020). The negative psychological effects of the COVID-19 pandemic may be linked to three psychological dimensions: (1) Increased distress, worry, and rumination (Limcaoco et al., 2020); (2) Consequences of quarantine and social isolation (Nitschke et al., 2021); (3) Reduction of positive experiences and emotions (Moroń and Biolik-Moroń, 2021).

Distress, worry, and rumination are core characteristics in the majority of mental illnesses and, more specifically, core features in generalized anxiety disorder and depression (Ehring and Watkins, 2008). Studies also suggest that stressful life events increase rumination, which further increases the risk of developing anxiety and depression (Michl et al., 2013). Treatment approaches like Cognitive Behavioral Therapy (CBT) and Metacognitive Therapy have been found to have a positive effect on both the treatment and prevention of worry and rumination (Topper et al., 2017).

Quarantine and social isolation have various consequences on individuals in different life situations. Negative psychological effects of quarantine include fear, fatigue, anger, irritability, grief, numbness, and sleep problems (Brooks et al., 2020). The psychological consequences of quarantine are affected by several components, such as duration of quarantine, no clear time limit of the quarantine period, fear of infection, frustration and boredom, lack of access to essential goods, and lack of information. Quarantine periods longer than reasonable based on the knowledge on incubation periods would increase the negative psychological effects (Brooks et al., 2020). One study found that participants who were in quarantine for longer than 10 days had more post-traumatic stress symptoms than those who were in quarantine for less than 10 days (Hawryluck et al., 2004). A large Norwegian epidemiological study including 10,084 adult participants also found that social distancing strategies during the COVID-19 pandemic were associated with increased self-reported symptoms of anxiety and depression (Ebrahimi et al., 2020). During the COVID-19 pandemic, the prevalence rate in the general population was estimated to 30.78% for depression and 27.57% for anxiety, whereas the prevalence rate of the same population before the pandemic in 2015 was 10.24% for depression and 14.70% for anxiety (Ebrahimi et al., 2020). For comparison, the prevalence rate in the general population in other countries in a non-pandemic period ranged from 6.10 to 10.80% for depression (Johansson et al., 2013; Maske et al., 2016; Kauffman et al., 2020) and 8.20 to 14.70% for anxiety (Johansson et al., 2013; Maideen et al., 2015; Hinz et al., 2017). Due to the negative psychological effects of long-lasting quarantine, social isolation, and social distancing, it is recommended that the general population receive help to maintain meaningful activity during pandemics (Brooks et al., 2020).

The reduction of positive experiences and emotions plays a vital role in the development and persistence of common mental health disorders (Moroń and Biolik-Moroń, 2021). Psychological prevention- and treatment programs have to a large degree focused on reducing negative emotions and functional impairments (Craske et al., 2016). At the same time, previous research shows that a lack of interest and pleasure from daily activities, which are the core symptoms of depression, are difficult to treat with the current treatment approaches (Craske et al., 2016). This might be due to the small or non-existent sense of reward that depressed individuals receive from their daily activities (Terry et al., 2020). Interventions with the aim to increase the attention to and expectancy of positive emotions have been shown to result in an increase in positive emotions (Craske et al., 2019). Moreover, behavioral activation, a psychotherapeutic intervention focused on increasing engagement in activities which increase coping and positive emotions, has also been found to be effective in reducing core symptoms of depression (Cuijpers et al., 2007).

Digital technology is a widespread and viable distributor of effective and scalable psychological interventions. Research and healthcare service innovations over the past 20 years have shown that digital technologies are effective methods for reaching more individuals with evidence-based psychological interventions (Andersson and Titov, 2014; Titov et al., 2018). Within mental health care, there are now hundreds of studies that document the effect of both therapist-guided and self-guided Internet-delivered interventions for mental illnesses like anxiety disorders, depression, and sleep disorders (Ebert et al., 2018; Karyotaki et al., 2018; Titov et al., 2018; Karekla et al., 2019). In light of the COVID-19 pandemic, self-guided Internet-delivered interventions may be a feasible way to provide psychological interventions, as they do not require face-to-face meetings, traveling, or any other form of physical contact (Karyotaki et al., 2017; Wind et al., 2020). In general, therapist-guided Internet-delivered interventions are associated with better treatment outcomes than self-guided Internet-delivered interventions, however, many people still benefit from a self-guided format. For instance, individuals with milder depressive symptoms experience similar benefits from therapist-guided interventions and self-guided interventions (Karyotaki et al., 2021). Another challenge with self-guided interventions for anxiety and depression is lack of sustained adherence, where the usage gradually declines with time, with between 21 and 88% of participants using the intervention in the beginning and between 0.5 and 28.7% completing the intervention (Fleming et al., 2018). Therefore, it is plausible that reducing time spent in treatment and offering a more concentrated treatment could help to improve completion rates. It is also important to note that there are some barriers when it comes to Internet-delivered interventions, as many elderly have lower technical iteracy than the younger generations (Kruse et al., 2020). Internet delivered interventions can also impose difficulties for vulnerable populations, such as those with impaired eyesight. However, as the COVID-19 pandemic adds more demand and strain on the healthcare system, self-guided interventions could be of extra benefit as they require less resources than other formats of delivery within mental healthcare (Karyotaki et al., 2017).

In this paper, we will first present the development of a self-guided psychological Internet-delivered intervention for adults experiencing increased distress during the first 9 months of the COVID-19 pandemic in Norway. Secondly, we will present between-group differences in clinical outcomes and adherence among participants receiving intervention modules at a 3-day interval and participants receiving intervention modules at a 5-day interval, as well as preliminary clinical effects for the total sample.

## Materials and Methods

### Study Design

The current study was a randomized-controlled trial (RCT, ClinicalTrials.gov: 126376). Using a randomizing generator through the website randomization.com, eligible participants were randomized to either (1) a new module every third day (3-day group, a total of 28 days) or (2) a new module every fifth day (5-day group, a total of 40 days). In this study, we present results from pre, post and follow-up measurements from the total sample of 82 participants.

### Ethical Approval

The study received ethical approval from the Norwegian Regional Ethical Committee (REK 126376) in an one week fast-track approval process as a response to COVID-19 related studies. Electronic informed consent was obtained from all participants.

### Setting

The intervention was developed in a cross-disciplinary and cross-sectorial team within the framework of the research project Introducing mental health through adaptive technology (INTROMAT, 259293), an ICT Lighthouse research project funded by the Norwegian Research Council, hosted by a University Hospital in Norway. In the INTROMAT-project, four Internet-delivered interventions, both therapist-guided and self-guided, were developed before the onset of the pandemic, and are currently being evaluated in multiple clinical trials. The developmental team included clinical psychologists, researchers in clinical psychology, Human-Computer Interaction, machine learning and computer modeling, and one industry partner.

### Development of the Intervention

The person-based approach (PBA; Yardley et al., 2015), a user-centered method for developing Internet-delivered interventions, served as a framework for the development of the current COVID-19 intervention. The PBA integrates knowledge and expertise from the research literature, theoretical approaches, and the end-users needs and preferences, applying both quantitative and qualitative methods in the planning, development, and evaluating phases of a new intervention (Yardley et al., 2015). In the present study we consulted the literature for possible effective intervention elements (Topper et al., 2017; Craske et al., 2019). The PBA recommends involving end-users to form guiding principles of the intervention development. However, due to the urgent need for an internet-delivered intervention, we opted to not include end-users in the development phase during a nationwide lockdown. Therefore, we revisited qualitative interviews conducted with individuals suffering from mental disorders in previous interventions in order to understand the potential needs and challenges of the target users. This formed the basis of the COVID-19 intervention’s guiding principles (Table 1), which state the design objectives and key features that address these. The guiding principles were used as a map to guide the development and adaptation of content, such as text and design features (Table 1).

**TABLE 1:** Guiding principles.

**Design objectives**	**Key features**
Create content targeting psychological distress (e.g., worries, rumination, consequences of isolation, reduction of positive emotions) associated with the COVID-19 pandemic.	Provide exercises based on evidence-based practice such as cognitive behavioral therapy, meta-cognitive therapy, and positive psychology.
Deliver content that provides hope and normalization.	Include quotes from other individuals affected by the COVID-19 pandemic. Use optimistic and calming pictures, such as photography of nature.
Provide content that is interesting and perceived as credible.	Provide informative knowledge that is scientifically funded and delivered from credible sources.
Provide content that is easy-to-understand.	Write material in an easy-to-understand language and deliver exercises that are easy to complete.

Based on the guiding principles, the majority of the content in the digital COVID-19 intervention was adapted from four previously developed digital interventions, all including systematic user-involvement and user-testing: (1) *MyADHD;* (2) *RestDep;* (3) *Gynea*, *and* (4) *YoungSpotlight. MyADHD* targets adults with ADHD with an aim to increase coping with everyday challenges related to ADHD. The intervention is self-guided, consists of seven modules, and builds upon principles from cognitive rehabilitation and mindfulness- and acceptance-based approaches. Preliminary results indicate improvement in self-reported symptoms of inattention and stress, and quality of life (Nordby et al., 2021; ClinicalTrials.gov NCT04511169). *RestDep* targets adults with cognitive residual symptoms after depression aiming to reduce the negative consequences, such as depression relapse. The intervention is guided, consists of ten modules, and is based on psychoeducation, attention training, and compensatory strategies. Preliminary results indicate reduction in residual cognitive symptoms and rumination (Myklebost et al., 2021). *Gynea* targets women who have had gynecological cancer with the aim to increase quality of life and adjusting to life after cancer treatment. The intervention is guided and consists of eight modules and includes psychoeducation, and mindfulness and self-compassion exercises (Sekse et al., 2021). The intervention is currently being evaluated for clinical effects (ClinicalTrials.gov NCT04414436). *YoungSpotlight* targets adolescents’ fear of public speaking with the short-term aim to reduce public speaking anxiety symptoms in the classroom and the long-term aim to prevent the onset of generalized social anxiety disorder. The intervention is self-guided and a combination of virtual reality exposure training and text-based modules. Preliminary results indicated a significant reduction in public speaking anxiety symptoms (Kahlon et al., 2019; ClinicalTrials.gov NCT04396392).

The first participant accessed the intervention on 4th of April 2020, as soon as the development of the first two modules were completed. The development of the next six modules were completed at least 5 days before the first participant reached that module (see Table 2 for overview of the modules).

**TABLE 2:** Overview of modules.

**Module**	**Theme**	**Tasks**
Module 1	• Introduction. • Psychoeducation on the connection between thoughts, feelings and behavior. • Introduction to breathing and positive feelings.	• Breathing exercises. • Identifying positive feelings. • Developing a positive activity plan.
Module 2	• Establishing habits. • Psychoeducation about the autopilot. • Introduction on how to find a balance in daily life during COVID-19. • Introduction to self-compassion. • Psychoeducation on positive feelings.	• Talking to yourself as you would to a friend. • Plan activities associated with positive feelings. • Accepting attitude toward thoughts and feelings.
Module 3	• Worries and inner tension. • Psychoeducation about worrying.	• Select activities associated with accomplishment and positive feelings.
Module 4	• Together and alone. • Psychoeducation about increasing self-compassion.	• Address difficult thoughts self-compassionately by writing kindly and supportive quotes. • Attention training. • Plan positive social activities.
Module 5	• New daily rhythm. • Introduction to planning and completing activities. • Psychoeducation about sleep hygiene, physical activity, and alcohol use.	• Develop to-do lists and sub-goals. • Sleep hygiene reflection task. • Write a positive activity plan.
Module 6	• Acts of kindness. • Psychoeducation about self-compassionate movements. • Introduction on how to strengthen relationships and emotional regulation.	• Compassionate touch and stretching. • Writing positive and compassionate thoughts toward a partner. • Time-out to cope with difficult feelings. • Gratitude exercise.
Module 7	• Psychoeducation about rumination and worries.	• Identifying rumination and worries. • Planning and register strategies to cope with rumination (e.g., thoughts pass; office time for ruminative thoughts and worries).
Module 8	• Planning the future. • Recap of modules.	• Review previous material. • Planning future strategies and activities for further coping of distress related to COVID-19.

### Designing and Evaluating the Participant’s Front-End

The development of the intervention was enabled by a digital platform for delivering interventions, previously developed in collaboration with the industry-partner Youwell AS as part of the INTROMAT project. The platform has three front-ends for different means that can be used in an Internet browser: (1) The participant’s front-end for accessing and using the intervention; (2) the therapist’s front-end for monitoring participants’ progress; (3) and an authoring tool for creating and modifying content and customizing the work-flow of the intervention.

The interface design of the participant’s front-end can be customized to any Internet-delivered intervention including idiosyncratic workflows, informed consents and randomization procedures. It is also possible to customize the participant’s front-end for different participant groups in each Internet-delivered intervention. All front-ends of the platform require two-factor authentication. However, it is also possible to make certain modules of the Internet-delivered programs publicly available. This was done to distribute information about the intervention, such as inclusion criteria, information about the research project and eligibility screening for people interested in participating in the trial.

The platform is structured in a hierarchy of programs, modules, pages and tasks. Text, pictures, audio, video, worksheets, and questionnaires can easily be developed and edited by non-technical personnel. The platform can also push automated prompts and reminders to the participant, and it collects and stores health- and interaction-data that may be used for clinical and IT-related research.

Internet-delivered interventions should be fully functional and usable to the general public on all Internet browsers and devices (laptops, tablets, smartphones) (Yogarajah et al., 2020). It should also be accessible to persons with disabilities. To reach this goal, we concurrently performed formal and informal usability evaluations of the participant’s front-end with developing the program content. Evaluation types included: functional testing with predefined tasks (Bruun and Stage, 2012), accessibility evaluation (Mankoff et al., 2005), and *ad hoc* usability testing (Følstad et al., 2013).

The functional testing was done by pre-defining tasks with descriptions of expected results to be completed within the participant’s front-end of the intervention platform (Bruun and Stage, 2012). Two persons piloted the tests simultaneously and described the results from their tasks in a spreadsheet. In the case of functional failures, these were described and corrected.

Accessibility evaluations were done according to Web Content Accessibility Guidelines (WCAG) 2.0 (Caldwell et al., 2008) and specifications by the World Wide Web Consortium (W3C). The guidelines specify recommendations for making websites accessible for people with disabilities. Web accessibility is protected by law through the EU Web Accessibility Directive and the Norwegian Digitalization Agency. Two persons conducted an accessibility evaluation (Mankoff et al., 2005). A third person then compared the results, compiled a list of accessibility issues and handed these over to the industrial partner who developed and maintained the platform. Due to the time constraints of rapidly answering to the COVID-19 situation, these accessibility tests were done *ad hoc*. Proper tools for assessing and exploring website accessibility (a screen reader extension and Chrome Developer Tools) were used in a Chrome web browser. Whenever a part of the participant’s front-end did not meet the WCAG 2.0 requirements, which is the level required by the Norwegian Digitalization Agency for public web sites, evaluators made a note detailing the shortcoming. For example, the sequence of elements in questionnaires had to be rearranged to be made accessible for visually impaired users.

Finally, *ad hoc* usability testing (Følstad et al., 2013) was done while developing the intervention. In this activity, we noted all front-end usability errors and faults that could negatively affect the user experience. These usability tests were organized by email and the chat service Slack.

**FIGURE 1:**
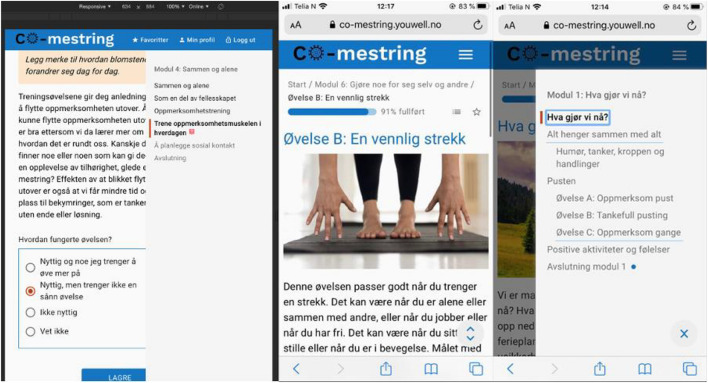
Illustration of the program website.

Based on these evaluations, the interface design of the participant’s front-end was improved by iterative development so that it would be fully responsive to different devices (both stationary and mobile). Existing interface components in the platform were improved for better usability and accessibility (see Figure 1 for an example). New interface components were rapidly sketched and prototyped in the web languages HTML and CSS. Sketches of designs and excerpts of HTML and CSS code were then handed over to the industrial partner who developed and managed the platform, and they in turn implemented the specified designs in the front-end. Following these improvements, the participant’s front-end complied with WCAG 2.0 requirements, and functional testing was completed without errors on all devices.

### Recruitment and Procedure

The participants were recruited through social- and traditional media from all over Norway. Interested participants were first directed to an open website with information about the study and an online screening to evaluate eligibility according to the following inclusion criteria: (1) 18 years of age or older, (2) self-reported mild and moderate distress in relation to the COVID-19 pandemic, (3) a score on the Patient Health Questionnaire between 5 and 14 (PHQ-9; Kroenke et al., 2001), and (4) able to read and write in Norwegian. The exclusion criteria were (1) currently receiving psychological or somatic treatment and (2) self-reported severe mental disorder, such as psychosis or substance abuse disorder. Eligible participants were invited to leave their contact information to be contacted by the study team for a phone screening interview. A clinical psychologist or psychiatric nurse conducted the screening interviews for further evaluation and obtained the national identity number of the eligible participants. Eligible participants then were randomized to a treatment group and thereby asked to digitally sign an informed consent before they were given access to the pre-treatment assessment and first treatment module.

### Outcome Measures

#### Primary Clinical Outcome Measure

The Patient Health Questionnaire (PHQ-9; Kroenke et al., 2001) measures depression and consists of nine items rated on a four-point scale scored 0 (“not at all”) to 3 (“nearly every day”). A short version of PHQ-9 with the first two items was used in the weekly measurements. A higher score indicates greater depression severity. Cronbrach’s alpha for PHQ-9 for this sample was 0.58. The low value is partly explained by the fact that PHQ assesses all dimensions of depression, a very heterogeneous disorder.

#### Secondary Clinical Outcome Measures

Generalized Anxiety Disorder 7 (GAD-7; Spitzer et al., 2006) measures the severity of general anxiety and consists of seven items rated on a four-point scale ranging from 0 (“not at all”) to 3 (“nearly every day”). A short version of GAD-7 with the first two items was used in the weekly measurements. Cronbrach’s alpha for GAD-7 for this sample was 0.80.

The positive and negative affect scale (PANAS; Watson et al., 1988) measures the positive and negative effect experienced and consists of ten adjectives related to emotions rated on a five-point Likert scale ranging from 1 (“very slightly/not at all”) to 5 (“very much”). The total sum score of both positive and negative affect is then calculated. In the current sample, Cronbach’s alpha for the positive affect scale was 0.87 and for the negative affect scale it was 0.74.

#### Treatment Satisfaction

Each module was evaluated by the participants with the following questions: (1) “To what extent do you find this module useful?” ranging from 1 = not useful at all, 10 = very useful; and (2) “To what degree of certainty would you recommend this module to a friend?” ranging from 1 = not certain at all, 10 = very certain. Participants also provided written feedback at the end of each module on the following questions: (1) “What did you like the most or what did you find as most useful in this module?,” and (2) “What did you miss or what disappointed you in this module?”

In addition, the Client Satisfaction Questionnaire (CSQ-8; Attkisson and Greenfield, 1994), an eight-items self-report questionnaire, was used to assess the overall satisfaction of the service on a scale from 1 = Not satisfied to 4 = Very satisfied.

### Statistical Analysis

SPSS Statistics 26 was used to perform the statistical analyses. Intention to treat analyses (ITT) of the treatment outcomes from pre-, post-, and follow-up assessments were performed using linear mixed models including random effects and random intercept. PHQ, GAD, PANAS positive, and PANAS negative served as the dependent variables. Missing data were handled according to Chakraborty and Gu (2009), as they were estimated and not imputed, by using restricted maximum likelihood (RML). This is assumed to affect the power of a clinical trial to a lesser degree compared to other strategies such as Last Observation Carried Forward or imputation for handling missing data (Chakraborty and Gu, 2009). All items in the PHQ-9 (primary measure) and GAD-7 and PANAS were assessed at pre-treatment, post-treatment, and 6 weeks follow-up, leaving three data points for the Mixed Linear Model analysis.

Effect sizes calculated as Cohen’s *d* were based on both observed and estimated means from pre-treatment to post- and follow-up treatment. Pre_*m*_ − Post_*m*_/SD_*pre*_. Standard deviation was calculated by standard error x√n.

As this randomized controlled trial had two relatively similar active arms and no previous relevant data were identified, no power calculator for between groups effects were conducted prior to the study. However, a *post hoc* power analysis was conducted.

## Results

### Participants

**FIGURE 2:**
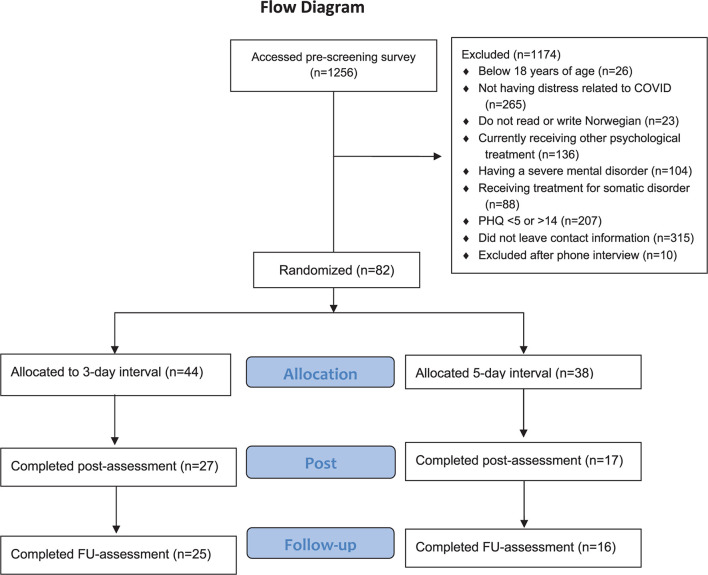
Flow Chart.

The recruitment started April 4th, 2020 and ended on December 14th, 2020. In this period a total of 1256 individuals accessed the pre-screening survey (see the Flow Chart in Figure 2), 407 were eligible according to the online screening, 92 provided contact information.

A total of 82 individuals were eligible to participate in the study and signed informed consent. The sample had a mean age of 40 (20–80, *SD* = 14.19). A total of 44 participants were randomized to the 3-day group and 38 participants to the 5-day group. Participant characteristics are shown in Table 3.

**TABLE 3:** Participant characteristics (*N* = 82).

	***n***	***%***
**Gender**		
*Female*	65	79%
*Male*	17	21%
Married or living with a partner	45	55%
Living alone	37	45%
Has children	43	52%
**Employment**		
*Full-time employed/student*	61	74%
*Sick leave/disability pension*	10	12%
*Temporarily laid-off/work-assessment allowance*	8	10%
*Retired*	3	4%
**Education**		
*High school/vocational school*	15	18%
*College/University*	67	82%
Previously received psychological treatment	36	44%
Worries about themselves, a close friend, or family member contracts COVID-19	64	78%
Themselves, a close friend, or family member have had COVID-19.	4	5%

### Adherence

A total of 43 out of 82 participants (52.4%) completed the post-measurements, and 42 out of 82 participants (51.2%) completed the 6 weeks follow-up assessment. In terms of module completion, there was no significant difference between the 3-day group (*M* = 4.91, *SD* = 3.07) and 5-day group (*M* = 4.42, *SD* = 3.02) group; [*t*(90) = 0.72, *p* = 0.472]. An overview of completed modules shows that 29 of the participants (66%) in the intensive group completed four modules or more, with 19 participants (43%) completing all eight modules, whereas in the 5-day group, 21 of the participants (55%) completed four modules or more, with 14 participants (37%) completing all eight modules. For the total sample (*n* = 82), 50 participants (61%) completed four modules or more, and 33 participants (40.2%) completed all modules.

### Clinical Outcomes

No significant between-group differences were identified between the 3-day and 5-day group (Table 4).

**TABLE 4:** Module completion among participants.

**Module**	**All participants *n* = 82**		**3-day group *n* = 44**		**5-day group *n* = 38**	
	**Started**	**Completed**	**Started**	**Completed**	**Started**	**Completed**
1	78	74	42	39	36	35
2	73	62	39	33	34	29
3	64	53	35	30	29	23
4	56	50	33	29	23	21
5	49	43	28	26	21	17
6	48	35	29	21	19	14
7	45	34	26	19	19	15
8	37	33	21	19	16	14

The following analyses were conducted for both groups combined in order to enhance power.

Overall, the observed means shows small and moderate within effect sizes (Table 5). Estimated means and effect sizes are reported in Table 6. The intention-to-treat analyses were as follows.

**TABLE 5:** Observed Means, Standard Deviations, and Effect sizes for Primary and Secondary Outcome Measures at Pre, Post, and Follow-up.

		**All participants**				**5-day group**				**3-day group**			
**Measure**	**Assessment**	***n***	***M***	***SD***	***ES pre-post_*w*_ pre-FU_*w*_***	***n***	***M***	***SD***	***ES pre-post_*w*_ pre-FU_*w*_***	***n***	***M***	***SD***	***ES pre-post_*w*_ pre-FU_*w*_***
PHQ	Pre	82	9.23	3.08		38	9.53	3.25		44	8.98	2.95	
	Post	43	7.65	4.11	0.51	16	7.06	3.00	0.76	27	8.00	4.66	0.33
	Follow up	42	8.40	5.59	0.27	17	8.06	5.49	0.45	25	8.64	5.75	0.12
GAD	Pre	82	9.40	3.95		38	9.63	3.40		44	9.20	4.39	
	Post	43	6.23	4.04	0.80	16	5.75	3.80	1.14	27	6.52	4.22	0.61
	Follow up	42	7.67	4.76	0.44	17	7.94	4.51	0.50	25	7.48	5.01	0.39
PANAS	Pre	82	15.00	3.55		38	15.00	3.58		44	15.00	3.57	
Pos	Post	42	15.86	3.43	0.24	16	16.56	3.39	-0.44	26	15.42	3.45	0.12
	Follow up	41	16.29	4.27	0.36	16	17.31	4.47	-0.65	25	15.64	4.10	0.18
PANAS	Pre	82	14.16	3.71		38	14.16	3.18		44	14.16	4.15	
Neg	Post	42	11.43	3.56	0.74	16	11.38	3.30	0.87	26	11.46	3.77	0.65
	Follow up	41	11.34	4.07	0.76	16	12.06	4.42	0.66	25	10.88	3.85	0.79

*ES = Within-group Effect Size; FU = Follow-up; PHQ = Patient Health Questionnaire; GAD = General Anxiety Disorder; PANAS-Pos = Positive Emotion Subscale from the Positive and Negative Affect Schedule; PANAS-Neg = Negative Emotion Subscale from the Positive and Negative Affect Schedule.*

**TABLE 6:** Estimated means values and effect sizes.

	**Measure**	**Pre**		**Post**		**Follow-up**		**ES pre-post_*w*_ pre-FU_*w*_**
		**M (SD)**	**95% CI**	**M (SD)**	**95% CI**	**M (SD)**	**95% CI**	
All participants	PHQ	9.23 (4.07)	8.35–10.12	7.54 (5.34)	6.37–8.71	8.41 (5.43)	7.23–9.60	0.42 0.20
	GAD	9.40 (4.17)	8.49–10.32	6.63 (5.25)	5.49–7.76	8.04 (5.25)	6.90–9.19	0.67 0.33
	PANAS pos	15.00 (3.71)	14.19–15.81	16.08 (4.53)	15.08–17.07	16.29 (4.62)	15.29–17.29	0.29 0.35
	PANAS neg	14.16 (3.80)	13.33–14.99	11.35 (4.98)	10.26–12.44	11.43 (5.07)	10.33–12.53	0.74 0.72
5-day group	PHQ	9.53 (4.07)	8.22–10.84	6.96 (5.98)	5.04–8.87	8.18 (5.79)	6.31–10.04	0.63 0.33
	GAD	9.63 (4.19)	8.28–10.99	6.11 (5.67)	4.29–7.93	8.44 (5.55)	6.65–10.22	0.84 0.28
	PANAS pos	15.00 (3.70)	13.80–16.20	15.67 (4.01)	14.38–16.96	17.17 (4.87)	15.61–18.73	-0.18 -0.19
	PANAS neg	14,16 (3.82)	12.93–15.39	11,01 (5.49)	19.26–12,76	11,97 (5.49)	10,22–13.72	0.68 0.80
3-day group	PHQ	8.98 (4.11)	7.76–10.19	7.86 (5.04)	6.36–9.36	8.55 (5.24)	7.00–10.10	0.27 0.10
	GAD	9.21 (4.25)	7.94–10.47	6.89 (4.97)	5.42–8.37	7.75 (5.11)	6.24–9.27	0.54 0.34
	PANAS pos	15.00 (3.71)	13.89–16.11	15.67 (4.31)	14.38–16.96	15.71 (4.38)	14.40–17.02	-0.18 -0.59
	PANAS neg	14,16 (3.85)	13.02–15.30	11,54 (4.71)	10.13–12–95	11,09 (4.78)	9.66–12.52	0.82 0.57

**ES* = Within group Effect Size; PHQ = Patient Health Questionnaire; GAD = General Anxiety Disorder; PANAS-Pos = Positive Emotion Subscale from the Positive and Negative Affect Schedule; PANAS-Neg = Negative Emotion Subscale from the Positive and Negative Affect Schedule.*

#### Depressive Symptoms

The linear mixed model showed a significant decrease in PHQ-9 from pre to post *(p* < 0.05), with an effect size of 0.42 (Tables 6, 7). However, when comparing pre with follow up measurement, the decrease in symptoms was not significant (*p* = 0.201).

**TABLE 7:** Intention to treat analyses from pre to post, and pre to follow up.

	***SE***	***df***	***t***	***p***	***CI***
**All participants**					
PHQ					
*Pre to post*	0.63	113.10	-2.67	<0.05	-2.95 – -0.44
*Pre to follow up*	0.64	113.36	-1.29	0.201	-2.09–0.44
GAD					
*Pre to post*	0.53	96.60	-5.2	<0.001	-3.84 – -1.72
*Pre to follow up*	0.54	96.68	-2.53	<0.05	-2.43 – -0.29
PANAS pos					
*Pre to post*	0.44	92.44	2.44	<0.05	0.20–1.96
*Pre to follow up*	0.45	92.49	2.88	<0.05	0.40–2.17
PANAS neg					
*Pre to post*	0.56	97.91	-4.99	<0.001	-3.93 – -1.69
*Pre to follow up*	0.57	98.09	-4.80	<0.001	-3.86 – -1.60
**5-day group vs. 3-day group**					
PHQ					
*Pre to post*	1.31	112.21	1.11	0.269	-1.14–4.04
*Pre to follow up*	1.30	112.12	0.71	0.482	-1.66–3.50
GAD					
*Pre to post*	1.10	95.09	1.10	0.273	-0.97–3.40
*Pre to follow up*	1.10	95.06	-0.23	0.817	-2.43–1.92
PANAS pos					
*Pre to post*	-1.03	90.59	-1.14	0.259	2.83 – -0.78
*Pre to follow up*	-1.46	90.61	-1.60	0.113	-3.27–0.35
PANAS neg					
*Pre to post*	0.53	96.43	0.45	0.651	-1.78–2.83
*Pre to follow up*	-0.89	96.53	-0.76	0.449	-3.20–1.43

*PHQ = Patient Health Questionnaire; GAD = General Anxiety Disorder; PANAS-Pos = Positive Emotion Subscale from the Positive and Negative Affect Schedule; PANAS-Neg = Negative Emotion Subscale from the Positive and Negative Affect Schedule.*

#### Anxiety Symptoms

The linear mixed model showed a significant decrease in GAD-7 from pre to post *(p* < 0.001) and from pre to follow up (*p* < 0.05) with an effect size of 0.67 and 0.33 (Tables 6, 7).

#### Positive and Negative Affect

The linear mixed model of the PANAS showed a small but significant increase in positive emotions from both pre to post (*p* < 0.05) and from pre to follow up *(p* < 0.05), with an effect size of 0.29 and 0.35 (Tables 6, 7), and a significant decrease in negative emotions over time from both pre to post (*p* < 0.001) and from pre to follow up *(p* < 0.001), with effect sizes 0.74 and 0.72 (Tables 6, 7).

### Treatment Satisfaction

Usefulness of the modules was rated between 6.58 and 7.65 for all participants. When asked if they would recommend the module to a friend, the participants gave a score between 6.74 and 7.85 (Table 8). Participants generally offered positive feedback on the content, and most participants mentioned the practical exercises (e.g., breathing exercises and activity planning) as the most useful elements in the modules. Few participants provided feedback on missing or disappointing elements. On the Client Satisfaction scale, the participants (*n* = 41) gave the intervention an overall rating of 2.60–3.34 (Table 9).

**TABLE 8:** Satisfaction of each module.

**Module**	**All participants *n* = 82**		**3-day group *n* = 44**		**5-day group *n* = 38**	
	**Usefulness**	**Recommend to a friend**	**Usefulness**	**Recommend to a friend**	**Usefulness**	**Recommend to a friend**
	**M (SD)**	**M (SD)**	**M (SD)**	**M (SD)**	**M (SD)**	**M (SD)**
1	7.11 (1.64)	7.57 (1.64)	7.49 (1.55)	7.79 (1.58)	6.69 (1.66)	7.31 (1.69)
2	6.58 (2.00)	6.74 (2.42)	6.94 (2.05)	7.18 (2.31)	6.17 (1.89)	6.24 (2.49)
3	7.32 (2.07)	7.34 (2.25)	7.80 (2.12)	7.80 (2.19)	6.70 (1.87)	6.74 (2.24)
4	7.06 (1.75)	6.90 (2.07)	7.52 (1.57)	7.45 (2.01)	6.43 (1.83)	6.14 (1.96)
5	6.58 (2.45)	6.93 (2.63)	6.73 (2.63)	7.23 (2.66)	6.35 (2.21)	6.47 (2.60)
6	7.49 (1.77)	7.69 (1.92)	7.86 (1.62)	8.05 (1.83)	6.93 (1.90)	7.14 (1.99)
7	7.65 (1.69)	7.85 (1.88)	7.68 (1.77)	8.05 (1.81)	7.60 (1.64)	7.60 (1.99)

*Usefulness ranges from 1 (not useful at all) to 10 (very useful); Recommend to a friend ranges from 1 (not likely at all) to 10 (very likely).*

**TABLE 9:** Client Satisfaction Questionnaire (*n* = 41).

**Question**	***M***	***SD***
1. Quality of service?	3.05	0.67
2. Kind of service you wanted?	3.00	0.55
3. Extent the program met your needs?	2.60	0.82
4. Recommend program to friend?	3.34	0.66
5. Satisfaction with the amount of help received?	3.02	0.61
6. Services helped you to deal with problems?	3.10	0.54
7. Overall satisfaction with the service?	3.15	0.62
8. Return to program for help?	3.27	0.67

*Satisfaction ranges from 1 (not satisfied) to 4 (very satisfied).*

### *Post hoc* Power Analysis

The *post hoc* power analysis showed that with a probability level of 0.05 with two groups with 44 and 38 participants the study was underpowered to detect significant differences between the two groups.

## Discussion

This study reports on the development and the clinical effects of a self-guided Internet-delivered intervention targeting psychological distress during the first months of the COVID-19 pandemic in Norway. In particular, the study assessed whether the intervention was associated with a reduction in depression symptoms, anxiety symptoms, negative emotions, and increased positive emotions. Overall, 82 participants were included in the study and 43 completed the intervention. A relatively large proportion of the sample (36 of 82) had previous experience with psychological treatment. No difference was found between the groups receiving the intervention modules every third or fifth day. When examining the overall clinical effects, the linear mixed model analyses showed significant reductions with moderate effects for depressive symptoms (*d* = 0.42), anxiety symptoms (*d* = 0.67), and negative emotions (*d* = 0.74), and a small significant increase (*d* = 0.29) was observed in positive emotions.

No between-group differences were identified between those who automatically received a new module every third day and those who automatically received a new module every fifth day. This could indicate that reducing the time spent in treatment does not have a negative effect on treatment outcomes. However, the lack of significant difference could also be a result of the sample being too underpowered to detect such a difference. Hence, more data concerning different time intervals for module delivery in this intervention is needed in order to contribute to knowledge regarding the optimal interval of automated self-guided psychological interventions.

Regarding clinical outcomes for the total sample, the findings showed a moderate decrease in depressive and anxiety symptoms during the intervention. This is a somewhat higher effect compared to previous studies of self-guided Internet-delivered interventions (Karyotaki et al., 2017). The effects were somewhat lower at the 6-week follow-up. The change from post to 6-week follow-up may be related to changes in the direct or indirect consequences of the pandemic. One factor may be uncertainty related to the duration of the pandemic and its consequences (Brooks et al., 2020). Another factor may be the increased attention on the negative economic impact of the COVID-19 pandemic, i.e., people becoming temporarily laid off, companies going bankrupt, or savings running out.

The findings showed a moderate and significant decrease in negative emotions over time, and a small but significant increase for positive emotions. The latter might be due to the challenge in finding activities that can increase positive emotions during quarantine. Activities that involve social interaction and physical contact are important for key positive emotions, and such activities were difficult to engage in during the pandemic (Moroń and Biolik-Moroń, 2021).

The adherence in the current self-guided intervention was relatively high, as 35 participants (38.5%) completed all eight modules. This is considerably higher compared to a study by Lillevoll et al. (2014) who explored the feasibility and effects of a self-guided mental health intervention for Norwegian youths where 8.5% of the eligible participants logged into the intervention and few completed all modules. However, the discrepancy could be a result of different target populations and recruitment procedures as adults participating in the current study themselves made contact for participation, while the youths in the Lillevoll et al. (2014) study were recruited through a survey conducted in Norwegian high schools.

Overall, the participants in the present study reported being generally satisfied with the intervention and all modules were rated as helpful. These findings indicate that the current intervention is perceived as acceptable by the target group and thus have the potential to support other people in their psychological distress during the COVID-19 pandemic and possibly future pandemics.

Regarding the recruitment, there was much interest for the intervention, as 1256 individuals accessed the screening. However, only 407 individuals were found eligible, 92 provided contact information to the study team and 82 were included to receive the intervention. The relatively high number of individuals that were not eligible could indicate that the inclusion criteria were too narrow. This may also have affected the treatment response as a floor-effect should be expected in a sample where people with moderate and high symptom levels were excluded (Bower et al., 2013). Still, including participants with higher levels of depressive symptoms might not be appropriate because of issues such as risk of suicide (Simon et al., 2013). This is in line with experts recommending that those having a risk of suicidality should be excluded from participation in self-guided interventions, or that guided elements should be included to monitor such symptoms (Sander et al., 2020). We suggest that future self-guided psychological interventions aiming to respond quickly to future or ongoing crises, such as COVID-19, should be considered for a wider range of the population, including those with moderate levels of psychological symptoms.

The current study has several limitations. The main limitation was that the study was underpowered for a two-arm randomized controlled trial with two active arms and therefore underpowered to detect a difference between the 3-day and 5-day group. Another limitation is the lack of a waitlist control group. It would have been beneficial to include a waitlist control group to investigate natural recovery in a period where time is important. For example, seasonal changes and the trajectories of the pandemic could affect the results. However, this was not implemented due to ethical considerations at a time with need for, and very limited access to, psychological interventions. Another limitation is the fact that only 82 out of 1256 individuals who accessed the online screening were granted access to the intervention. This disproportionate number of included participants indicates a highly selected sample based on the inclusion/exclusion criteria and the number of participants leaving their contact information. Importantly, this limits the generalizability of these findings to a larger population. Furthermore, Cronbrach’s alpha for PHQ-9 for this sample was 0.58. The low value is partly explained by the fact that PHQ assesses all dimensions of depression, a very heterogeneous disorder.

Another limitation is the high number of eligible individuals that did not leave their contact information in order to be included in the study. This could be a result of them not being motivated to participate or having enough information to commit to participation. This could be addressed by providing more detailed information about the intervention and strengthening the rationale for treatment on the project web-page where participants self-screened. More research is needed to understand the conversion rates of the number of people starting the pre-screening survey versus the number of people completing the survey. Understanding the factors involved in successful onboarding can be of benefit for future digital interventions.

Taken together, the results of our study indicate that the present self-guided intervention for adults experiencing psychological distress during the COVID-19 pandemic may lead to reduction in symptoms of anxiety and depression, as well as negative emotions. The majority of the participants were satisfied with the treatment and the overall adherence was good when compared to other self-guided interventions. The lack of significant differences between the 3-day and 5-day group may also indicate that a shorter treatment duration can still produce similar treatment outcomes. Moving forward, the present non-commercial COVID-19 intervention was made publicly available free of charge from 2021 *via* the national eHealth platform in Norway (helsenorge.no), where all inhabitants in Norway can access their public eHealth services. In this context, the login still includes two-factor authentication, but without screening and the inhabitants get access to the intervention immediately after registration. The intervention will be continuously adapted to the trajectories of the ongoing COVID-19 pandemic. The results of the present study can be applied in preparence and response for future pandemic crises where the development of digital interventions for handling psychological distress are needed.

## Data Availability Statement

The raw data supporting the conclusions of this article will be made available by the authors, without undue reservation.

## Ethics Statement

The studies involving human participants were reviewed and approved by Regional Committees for Medical and Health Research Ethics West. The patients/participants provided their written informed consent to participate in this study.

## Author Contributions

TN have lead the work with the development of the intervention, planned the study, and wrote the manuscript. SK have lead the work with the analyses and contributed to the development of the intervention, planned the study, and wrote the manuscript. SM have lead the work related to the person-based approach and have contributed to the development of the intervention, planned the study, and wrote the manuscript. EF have lead the work with the user-testing and contributed to the development of the intervention, and wrote the manuscript. EN have contributed to the development of the intervention, planned the study, and wrote the manuscript. All authors contributed to the article and approved the submitted version.

## Conflict of Interest

The authors declare that the research was conducted in the absence of any commercial or financial relationships that could be construed as a potential conflict of interest.

## Publisher’s Note

All claims expressed in this article are solely those of the authors and do not necessarily represent those of their affiliated organizations, or those of the publisher, the editors and the reviewers. Any product that may be evaluated in this article, or claim that may be made by its manufacturer, is not guaranteed or endorsed by the publisher.
